# Multi-modal and multi-scale clinical retinal imaging system with pupil and retinal tracking

**DOI:** 10.1038/s41598-022-13631-1

**Published:** 2022-06-10

**Authors:** Muhammad Faizan Shirazi, Jordi Andilla, Nicolas Lefaudeux, Claudia Valdes, Florian Schwarzhans, Marine Durand, Konstantinos Ntatsis, Danilo Andrade De Jesus, Luisa Sanchez Brea, Kiyoko Gocho, Josselin Gautier, Christina Eckmann-Hansen, Marie Elise Wistrup Torm, Abdullah Amini, Stefan Klein, Theo Van Walsum, Kate Grieve, Michel Paques, Michael Larsen, Pablo Loza-Alvarez, Xavier Levecq, Nicolas Chateau, Michael Pircher

**Affiliations:** 1 Center for Medical Physics and Biomedical Engineering, Medical University of Vienna, Waehringer Guertel 18-20, 1090 Vienna, Austria; 2 ICFO—Institut de Ciencies Fotoniques, The Barcelona Institute of Science and Technology, 08860 Castelldefels, Barcelona Spain; 3 Imagine Eyes, Orsay, France; 4 Biomedical Imaging Group Rotterdam, Department of Radiology and Nuclear Medicine, Erasmus MC, Rotterdam, The Netherlands; 5 CHNO Des Quinze-Vingts, INSERM-DGOS CIC 1423, 28 rue de Charenton, Paris, France; 6 Sorbonne Université, INSERM, CNRS, Institut de La Vision, 17 rue Moreau, 75012 Paris, France; 7 Rigshospitalet, Department of Ophthalmology, University of Copenhagen, Copenhagen, Denmark; 8 Department of Electronic Engineering, Faculty of Electrical and Computer Engineering, NEDUET, Karachi, 75270 Pakistan

**Keywords:** Medical imaging, Imaging and sensing, Eye diseases

## Abstract

We present a compact multi-modal and multi-scale retinal imaging instrument with an angiographic functional extension for clinical use. The system integrates scanning laser ophthalmoscopy (SLO), optical coherence tomography (OCT) and OCT angiography (OCTA) imaging modalities and provides multi-scale fields of view. For high resolution, and high lateral resolution in particular, cellular imaging correction of aberrations by adaptive optics (AO) is employed. The entire instrument has a compact design and the scanning head is mounted on motorized translation stages that enable 3D self-alignment with respect to the subject’s eye by tracking the pupil position. Retinal tracking, based on the information provided by SLO, is incorporated in the instrument to compensate for retinal motion during OCT imaging. The imaging capabilities of the multi-modal and multi-scale instrument were tested by imaging healthy volunteers and patients.

## Introduction

Scanning laser ophthalmoscopy (SLO) and optical coherence tomography (OCT) are two imaging modalities that are regularly used for diagnosis of retinal diseases in clinical routine^1,2^. SLO provides an en-face view of the fundus while OCT gives volumetric information on retinal tissue. Using OCT angiography (OCTA), a functional extension of OCT, the contrast of vessels and capillaries in the retina is enhanced^3–6^. For visualization of cellular structures in the human eye, aberrations introduced by imperfections of the optics of the eye need to be corrected. This can be achieved by utilization of adaptive optics (AO) in combination with SLO or OCT. The benefits of AO for high-resolution imaging in SLO and OCT have previously been discussed^7–10^. With this technology, visualization of various cell types such as photoreceptors^11–17^, retinal pigment epithelium cells^18–20^, and ganglion cells^21,22^ has been demonstrated.

Although AO assisted imaging has become a vital tool in the vision science and ophthalmology community to explore pathological changes at a cellular level, the bulky size, single imaging modality and complex operation of such instruments are limiting factors for clinical translation. In addition, the limited field of view, typically on the order of 1–2 degrees, decreases the applicability of this technology in clinical routine. Thus, scientific and engineering research have been focused on more compact designs of instruments and on implementations of multimodal functionality^23–28^. To address the limited field of view of AO, an OCT system was recently introduced that allows for acquisition of large field of view volumes and to optically zoom into regions of interest with AO assisted higher transverse resolution^29^. In addition to AO, the combination of OCT and SLO sometimes provides complementary information^28^ and is beneficial for correcting motion artefacts that are present in OCT volume scans because SLO images are recorded much faster than entire OCT volumes.

Retinal tracking in real time is specifically important for AO-OCT because, for this high magnification, motion artefacts are much more pronounced^25,30–32^. As aberrations strongly depend on the pupil position with respect to the instrument^33,34^, the subject or the instrument usually need to be permanently aligned in order to achieve good image quality.

In this work, we introduce a compact, multi-modal and multi-scale retinal imaging instrument with angiographic functional extension for clinical use. Although various groups have demonstrated different multimodal combinations of these technologies in the research lab context^24,25,27^, here we combine all of these capabilities (i.e. large and small field, AO, SLO, OCT, OCT-A, retinal and pupil tracking) in a single clinical-use device for the first time. The compact instrument incorporates three imaging modalities: OCT, OCTA and SLO. Imaging can be performed at multiple scales with varying fields of view and transverse resolutions. The large field of view modes represent OCT/SLO imaging with standard resolution (without AO correction), while the small field of view mode allows for an optically magnified view into regions of interest with high transverse resolution due to adaptive optics correction. The instrument is equipped with a pupil tracker that allows for automatic self-alignment of the system with respect to the eye. Thus, the instrument can be fully operated by a single user. In addition, a retinal tracker that is based on the information provided by the SLO is incorporated to minimize motion artefacts in the OCT volume scan. Residual motion is removed by post processing. The performance of the system has been tested in vivo by imaging healthy subjects and patients with eye diseases.

## Results

**Figure 1:**
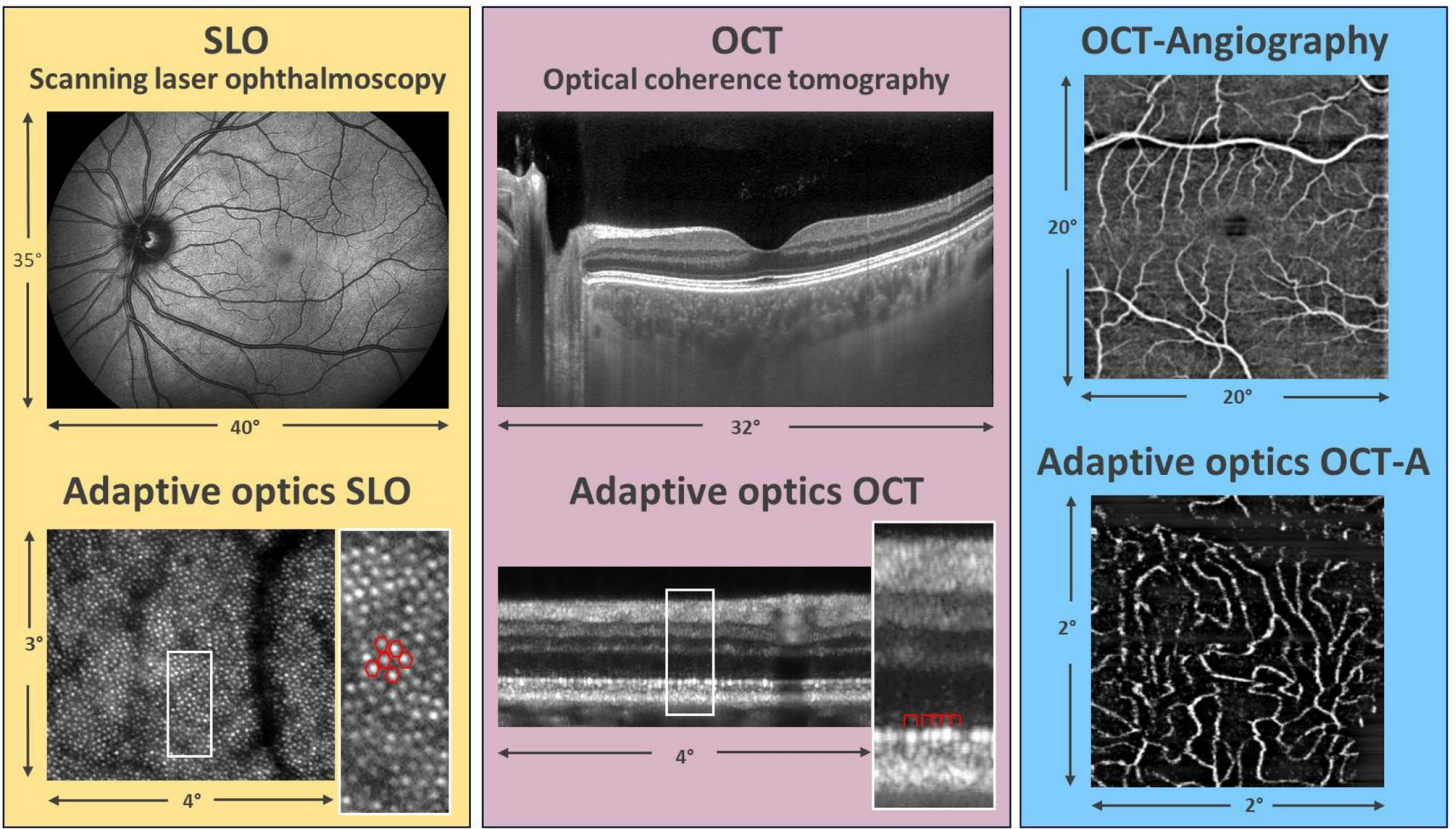
Overview of the multi-modal and multi-scale functionality of the instrument. The signal arising from individual cone photoreceptors is encircled in red color.

Figure 1 shows representative images of the multi-modal and multi-scale functionality of the instrument. The large field of view SLO image provides a good overview over the fundus of the eye, while the high resolution AO-SLO image shows cellular details such as the photoreceptor mosaic. Similarly, a large field of view OCT cross-sectional image shows the various retinal layers and high light penetration into the choroid, while the high-resolution AO-OCT image shows individual photoreceptors in the B-scan. Further, with the functional extension of OCT, angiographic images of the vascular network in the retina can be obtained with or without adaptive optics.

**Figure 2:**
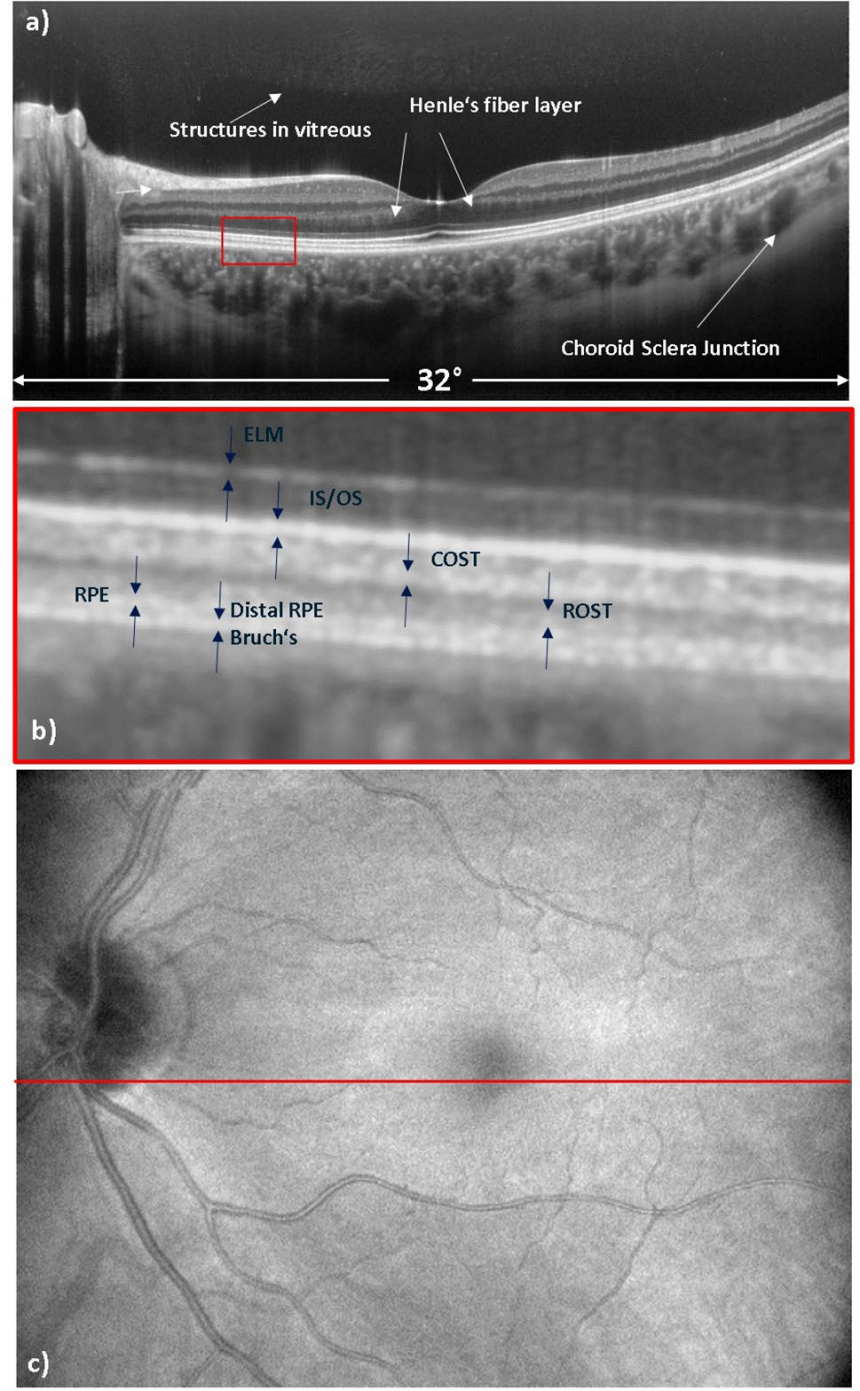
Representative OCT images recorded with the large field of view imaging mode in the healthy subject where (**a**) shows an average over 200 B-scans recorded at the same location, (**b**) shows an enlarged view of the posterior layers of the retina, and (**c**) shows the en-face intensity projection image of the volume scan. The red line at the center of the OCT en-face image indicates the location of the averaged B-scan image. ELM, external limiting membrane; IS/OS, inner and outer segment junction; COST, cone outer segment tips; ROST, rod outer segment tips; RPE, retinal pigment epithelium.

For a more detailed analysis of the imaging capabilities of the instrument, Fig. 2 shows representative large field of view imaging for OCT in the same subject as in Fig. 1. Figure 2a shows an average of 200 B-scans recorded at the same location on a logarithmic grey scale. The high signal to noise ratio in the image allows the visualization of structures in the vitreous humor of the eye. Because of the slight inclination of the retina with respect to the imaging beam^35^, Henle’s fiber layer is seen as a hypo-reflective band temporal to the fovea while it is hyper-reflective nasal to the fovea. Imaging at the 1060 nm wavelength band allows deep penetration into tissue and visualization of the choroidal-scleral junction as indicated with the arrows in Fig. 2a. Figure 2b is a zoomed image of the region indicated with the red rectangular box in Fig. 2a, which provides a better view of the different outer retinal layers. For the different layers in the retina we adapted the terminology as proposed by R. Jonnal et al.^36^. The posterior bands, the external limiting membrane (ELM), the junction between inner and outer segments of photoreceptors (IS/OS) and the cone outer segment tips (COST) appear as rather homogenous hyper-reflective bands over the entire image. In some works, the IS/OS and COST layers are referred to as ellipsoid zone and interdigitating zone, respectively^37^. At ~ 7–8° eccentricity from the fovea, we start to see an additional layer originating from the rod outer segment tips (ROST). As the rod outer segments are longer than the cone outer segments, the ROST are found posterior to the COST^19,38^. Posterior to the ROST layer, we observe the hyper-reflective layer of pebble-like granules that originates from the RPE, and then a similar, more posterior layer (distal RPE), which is attributed to the RPE and Bruch’s membrane^17^. Figure 2c is the large field of view en-face intensity projection image of the OCT with a 32° × 23° field of view.

**Figure 3:**
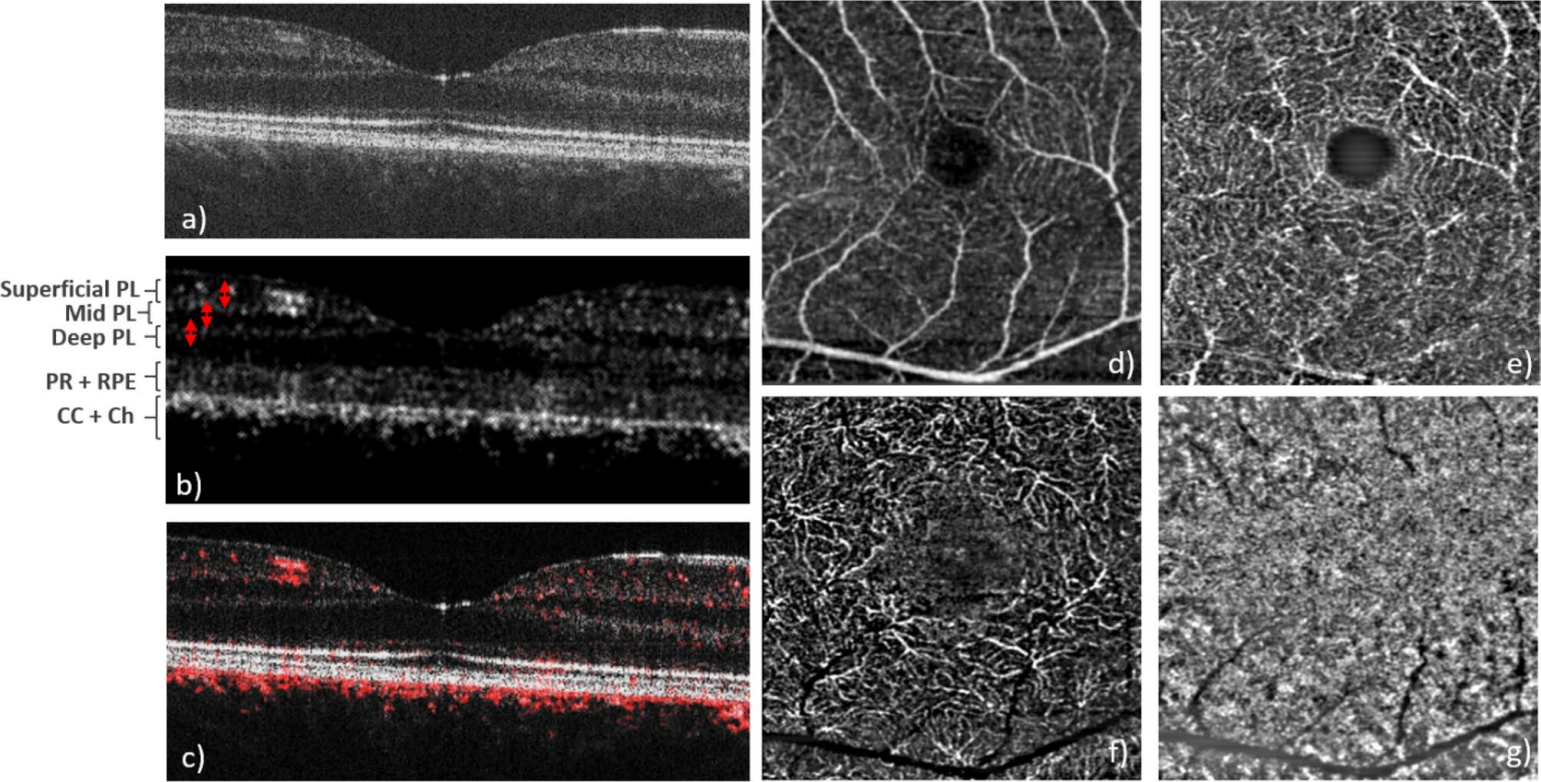
Representative image data recorded with the 10° × 10° field of view scanning pattern and 4 B-scans at the same location. (**a**) Single B-scan intensity image, (**b**) OCTA B-scan, (**c**) composite image of intensity (grey) and OCTA (red), (**d**) superficial vascular plexus, (**e**) mid vascular plexus, (**f**) deep vascular plexus (the integration depth of the en-face projections are indicated with the red arrows in **b**,**g**) choriocapillaris (en-face projection over three pixels below the RPE). *PL* plexus, *PR* photoreceptors, *RPE* retinal pigment epithelium, *CC* choriocapillaris, *Ch* choroid.

Figure 3 shows OCTA imaging results obtained within a 10° × 10° field of view and with the scanning pattern of 4 B-scans at each vertical position. In the intensity image (Fig. 3a) the retinal vasculature can hardly be seen. The angiographic data evaluation, based on motion-contrast, shows perfused blood vessels with high contrast (cf. Fig. 3b) because the signal from static tissue elements is suppressed. This is demonstrated by the preservation of the signal from the choriocapillaris vessels (CC), whereas the static OCT reflections from the photoreceptors (PR) and the pigment epithelium (RPE) are only faintly visible (Fig. 3b). The dynamic image is then overlaid on the static image and shown in red color on the composite image (Fig. 3c) and it is also used to compose an en-face view of the circulation of the choriocapillaris (Fig. 3g). The high reflectivity of the flowing blood in the choriocapillaris^39^ and corresponding reflectivity oscillations has the effect of reducing both static and dynamic OCT signals from the more posterior structures (Fig. 3a–c).

The en-face projections (cf. Fig. 3d–g) have been generated after segmentation of the ILM and depth integration of the data. The depth extension of each projection is indicated in Fig. 3b, and separates between superficial, mid, deep capillary plexus, and the choriocapillaris, similar to the angiographic evaluation of commercially available devices^40^. One specific asset of the device is the higher transverse optical resolution compared to commercial OCT devices. This results in an improved suppression of projection artefacts of anterior vessel layers, which is similar to observations made with AO-OCTA^41^.

**Figure 4:**
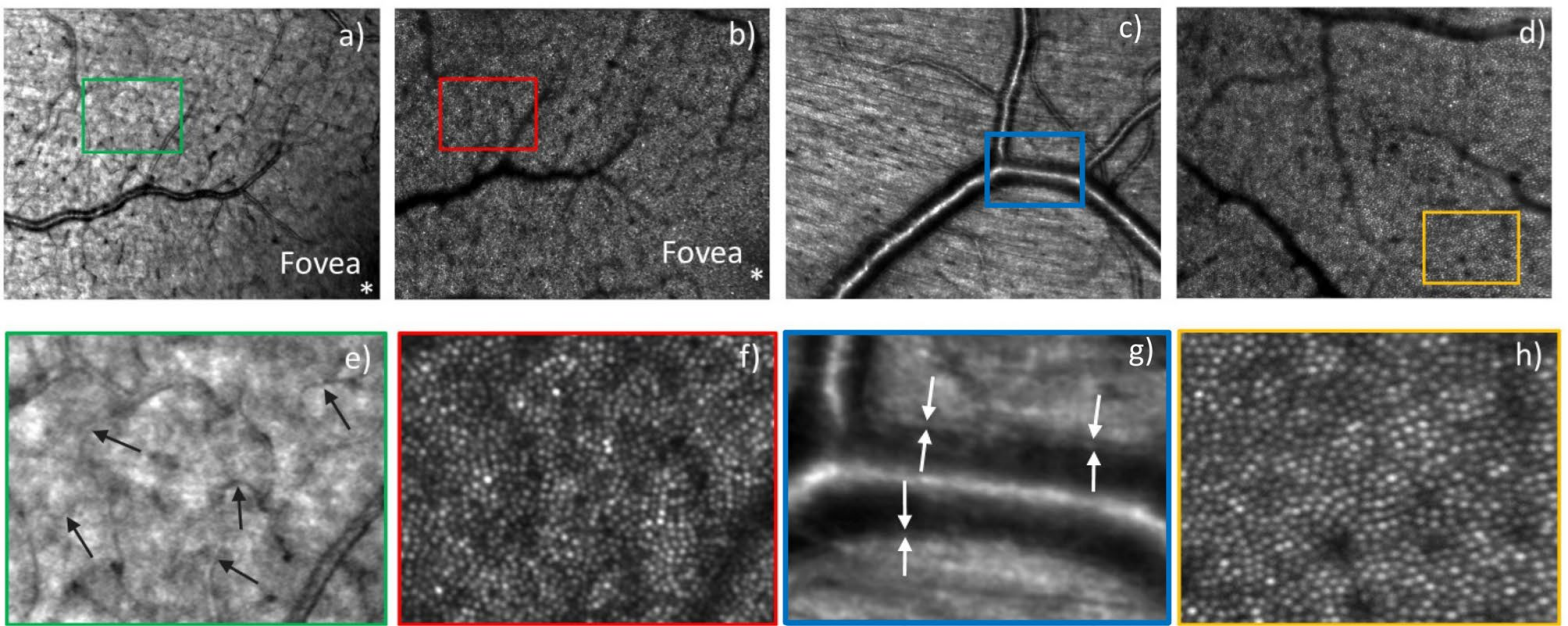
AO-SLO images of a healthy volunteer recorded with a field of view of 4° × 3°. (**a**) AO-SLO image from near the fovea with its focus on the nerve fiber layer and (**b**) the same field, now focused on the photoreceptor layer. Images (**c**,**d**) are from two different fields, centered at ~ 4° eccentricity from the fovea and focused on the nerve fiber layer and the photoreceptor layer, respectively. Figures (**e**–**h**) are the 4 × enlarged view of the rectangular boxes marked in Figures (**a**–**d**), respectively. The arrows in (**e**) indicate small capillaries while in (**g**) the vessel wall is marked.

Figure 4 shows high resolution AO imaging with SLO at different depths of focus and different eccentricities from the fovea, with a field of view of 4° × 3°. Figure 4a,b show the AO-SLO images near the fovea with a focus on the nerve fiber layer and photoreceptor layer, respectively, with the same region of interest. The images in Fig. 4c,d were recorded at two different locations centered at ~ 4-degree eccentricity from the fovea and focused on the nerve fiber layer and the photoreceptor layer, respectively. The nerve fiber bundles and fine vascular details such as capillaries and vessel walls in the nerve fiber layer can be distinguished. Figure 4f,h are the enlarged view (× 4) of photoreceptor cone mosaic features at ~ 3° eccentricity from fovea. At this eccentricity from the fovea, cone photoreceptors are visualized throughout the image. The fine capillaries and the vessel wall are indicated with arrows in the enlarged views (× 4) in Fig. 4e,g, respectively.

**Figure 5:**
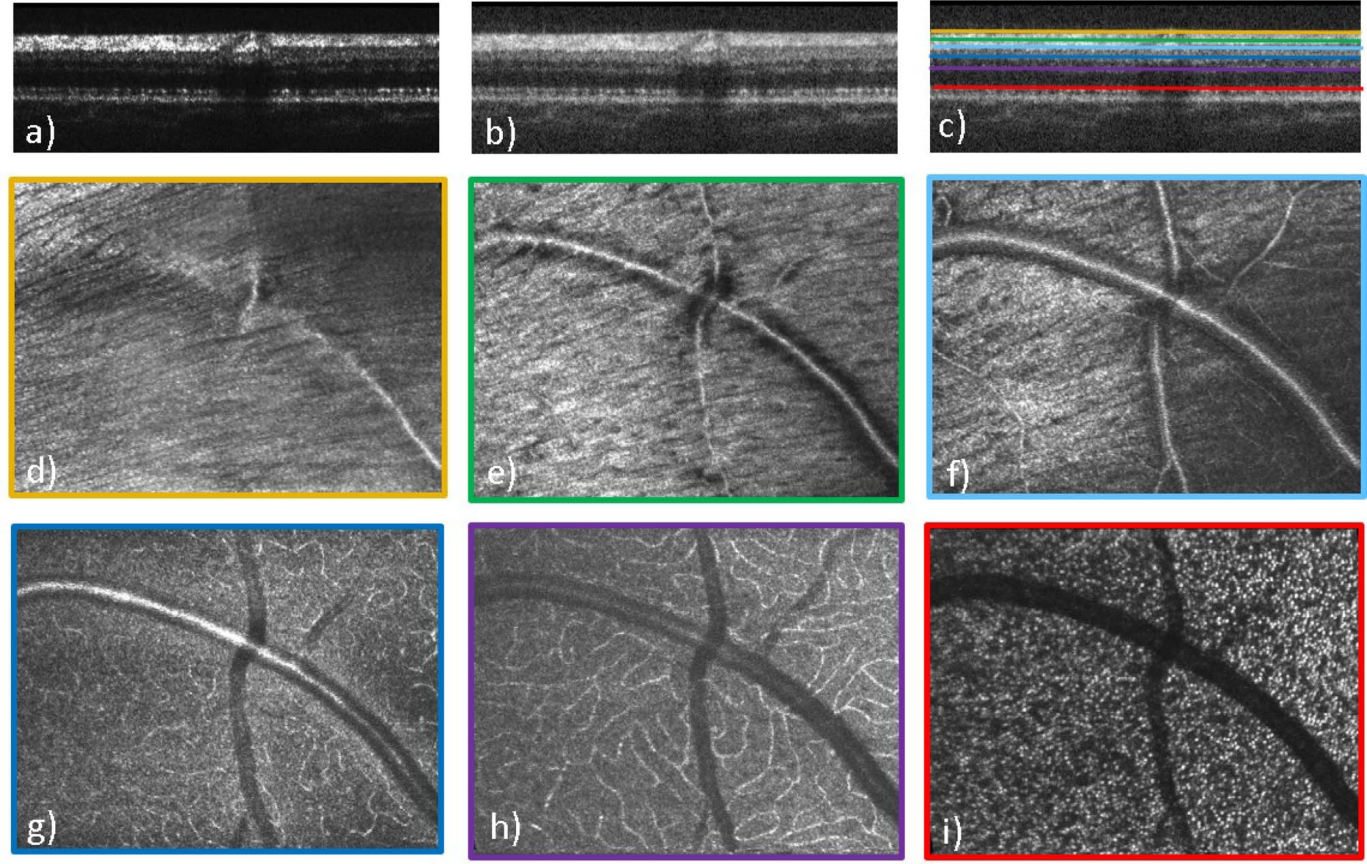
AO-OCT B-scan (**a**–**c**) and en-face images of different layers (**d**–**i**) recorded with a field of view of 4° × 3°. (**a**) B-scan in linear scale, (**b**) B-scan in log scale, (**c**) color-coded lines indicating the location of the corresponding en-face layers in B-scan, (**d**) inner limiting membrane, (**e**) nerve fiber layer, (**f**) superficial vascular plexus layer, (**g**) mid vascular plexus layer, (**h**) deep vascular plexus layer, (**i**) inner/outer segment junction layer.

Figure 5 shows high resolution AO-OCT images at ~ 8° diagonal eccentricity from the fovea at a superior temporal location. Figures 5a,b show the B-scan images in linear and log scales while Fig. 5c shows the axial position of different layers that are displayed in the corresponding en-face images. The focus of the AO was set to the nerve fiber layer. As a result, individual nerve fiber bundles can be clearly visualized (cf. Fig. 5d,e). Due to the increased numerical aperture of the imaging system, increased contrast at the capillaries can be seen to allow visualization of the different capillary plexuses with good sharpness. Although the focus is set to the anterior layers, reasonably sharp images of the photoreceptor mosaic can be observed at this eccentricity where the cone to cone spacing is ~ 9 µm. The 3D information provided by AO-OCT allows the visualization of a blood vessel crossing (cf. Fig. 5d–h) where the smaller, vertically oriented vessel lies on top of the larger, horizontally oriented one.

**Figure 6:**
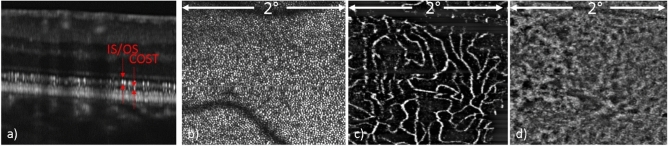
AO-OCT and AO-OCTA images of different layers with the field of view of 2° × 2°. (**a**) AO-OCT B-scan in logarithmic scale, (**b**) AO-OCT photoreceptor mosaic en-face image, (**c**) AO-OCTA deep plexus en-face image, (**d**) AO-OCTA en-face choriocapillaris image. *IS/OS* inner and outer segment junction, *COST* cone outer segment tips.

Figure 6 shows AO-OCT and AO-OCTA images of the fundus spanning a field of view of 2° × 2° with the focus set on the photoreceptor layer. An AO-OCT B-scan in logarithmic grey scale shows the layers of the retina at a resolution that allows identification of individual photoreceptors in the IS/OS and COST layers (Fig. 6a). An en-face AO-OCT image constructed from the stack of B-scans shows the cone mosaic pattern at a resolution that enables photoreceptor mapping and counting, with a small horizontal motion artifact near the middle of the image (Fig. 6b). An AO-OCTA slab from the deep capillary plexus (Fig. 6c) is shown in black-and-white for enhanced contrast, whereas the en-face choriocapillaris slab is shown without contrast enhancement (Fig. 6d). In comparison with images recorded by the large field of view mode, which is made without AO assistance, the individual capillaries are thinner and the interstitial spaces between the capillaries are wider, because finer imaging prevents the motion contrast signal to extend into areas where no motion occurs. This allows a more authentic rendition of capillary lumen diameters. Similarly, images of choriocapillaris (Fig. 6d) are much clearer than in the case of lower transverse resolution of the large field of view mode in Fig. 3g.

**Figure 7:**
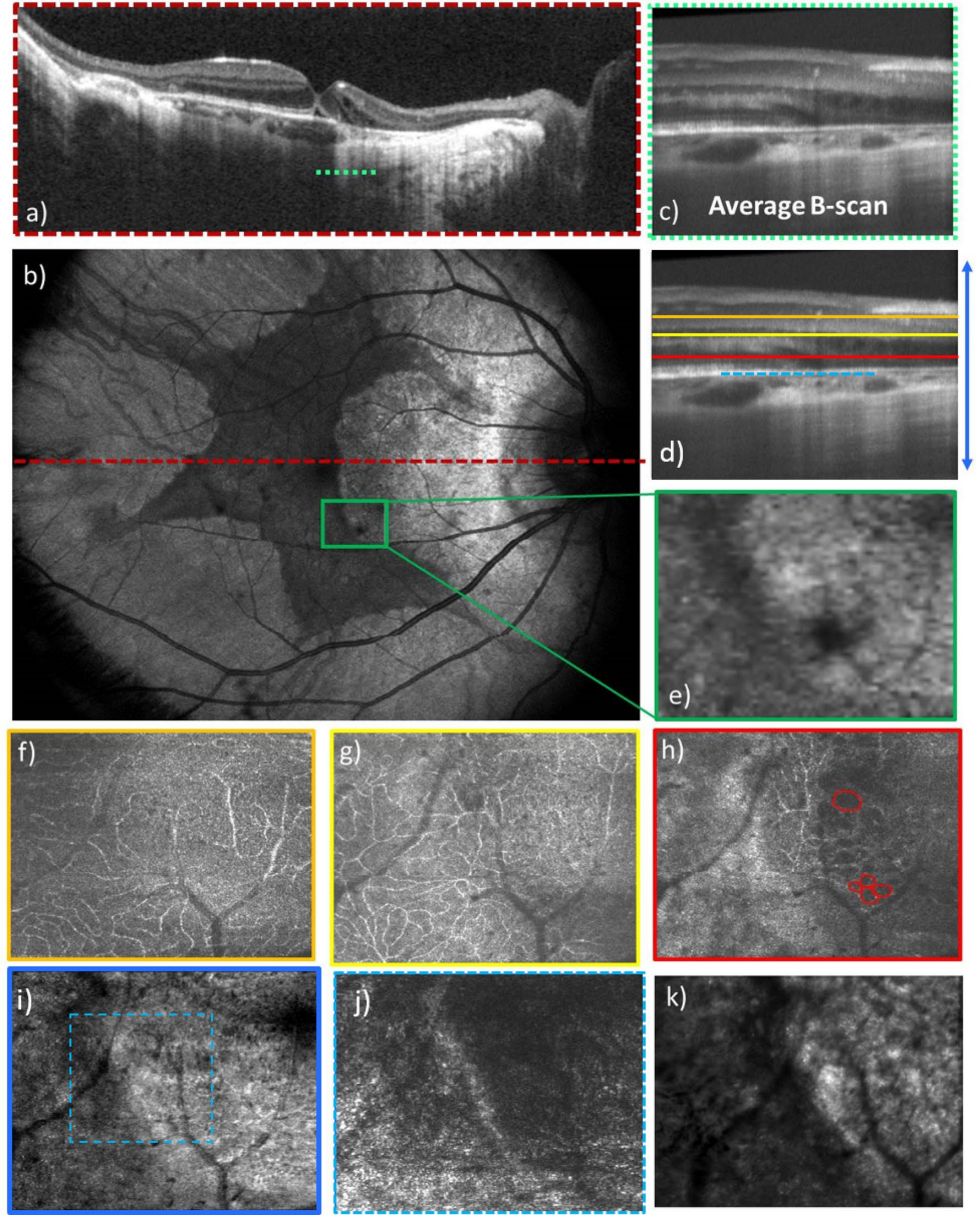
AO-OCT and AO-SLO images of a patient with choroideremia disease with 4° × 3° field of view along with the large field of view images. (**a**) Average OCT large field of view B-scan in log scale, (**b**) OCT large field of view en-face projection image with 40° × 30° field of view, (**c**) average AO-OCT B-scan in log scale, (**d**) location of corresponding layers displayed in the en-face images, (**e**) large field of view en-face projection image with zoom at the location imaged with AO-OCT, (**f**) mid capillary plexus layer, (**g**) deep capillary plexus layer, (**h**) outer nuclear layer with red circles indicating the microcysts, (**i**) entire depth projection image, (**j**) depth projection over photoreceptor and RPE layers at the 2° × 2° field marked in (**j**,**k**) AO-SLO image of the corresponding location.

In a final step, the instrument was tested in various patients. Figure 7 shows representative OCT, AO-OCT and AO-SLO images of a patient with a rare inherited retinal disease, choroideremia. The region of interest is indicated with a green rectangular box in the large field of view OCT en-face image in Fig. 7b. The large field of view B-scan image (Fig. 7a) shows a visualization of the sclera in the diseased region at the location shown with a red dashed line. The OCT en-face image of the same eye demonstrates distinct borders between the residual island of retinal tissue and the atrophic region. The AO-OCT B-scan image was created by averaging 10 adjacent B-scans in order to increase the signal to noise ratio. The different layers which are affected as a result of the chorioretinal atrophy are shown. The AO-OCT en-face images show the vasculature at different depths. In the outer nuclear layer, the retinal microcysts are surrounded by vasculature, as outlined in Fig. 7h. Similarly, the degeneration of photoreceptors and retinal pigment epithelium can be seen in the atrophic region in Fig. 7i. By comparing the AO-OCT images with AO-SLO and zoomed in OCT images, it is obvious that AO-OCT provides more insight about the disease at the region of interest.

## Discussion

A compact, multi-modal and multi-scale retinal imaging system that provides overview and high resolution images of different retinal regions has been developed and subjected to initial testing. With this single instrument, simultaneous SLO/OCT images can be obtained with and without correction of the aberrations by adaptive optics. A region of interest can be easily selected by a software interface on the large field of view SLO/OCT en-face images acquired prior to high resolution imaging. Overall, a multitude of images such as OCT, OCTA, SLO and AO-OCT, AO-OCTA, AO-SLO images are acquired during one single clinical session and provides a one-to-one correspondence between the various regions of interest that are imaged. The imaging capabilities of the instrument were demonstrated here by representative images of healthy subjects and of a patient with an inherited retinal disease, choroideremia.

In comparison with previously introduced compact instruments^27,29^ the new system incorporates significant modifications in order to further increase the usability and applicability of AO assisted instrumentations in clinical settings. One aspect is pupil tracking that greatly simplifies subject alignment and significantly reduces the overall time duration of an imaging session. One additional very important factor is that the instrument can be operated by a single user which opens the translation of AO assisted imaging into a clinical environment. For AO assisted imaging, a correct and stable alignment of the pupil with respect to the instrument is essential for obtaining good image quality. Simultaneous retinal tracking is another key element that has proven to be fast enough to compensate for drift and slow motion. The complete elimination of artefacts produced by fast motion occurring for example during saccades has yet to be accomplished, but with reasonable compliance from the test subject, the region of interest selected for high-resolution imaging by AO-OCT can be imaged to a level of practical utility and residual motion is then corrected by multimodal image registration software that uses the recorded SLO and AO-SLO images as reference for registration and alignment of the OCT and AO-OCT images, respectively.

The high transverse resolution of the large field of view OCT is certainly beneficial for angiographic evaluation of the data, as the backscattered intensity from the vessels is higher than in the case of low resolution imaging. This results in more pronounced contrast in the OCTA images. The AO assisted imaging mode allows in general for a better assessment of the underlying vascular structure, as small details can be visualized, which aids in the interpretation of the data acquired with the large field of view mode. We want to emphasize here that AO-OCTA, an extension that is not available to any OCT instrument on the market, provides a much better impression of the true extension of retinal vasculature and allows for visualization of the choriocapillaris network.

The current system, with a light exposure of ~ 1.3mW at the cornea, uses only about half the power of state of the art swept-source OCT instruments. This is partly because a conservative approach has been taken to safety calculations for the simultaneous operation of an SLO channel and an OCT channel, and because eye safety standards omit consideration of the constant rapid movement of the OCT and SLO imaging beam foci over the fundus. Safety calculations take into account the SLO and OCT scanning on the retina, and the exposures are calculated for the very short exposure time of the scanning motion when the beam passes over a single point of the retina. The power was reduced to cope with failure modes of the system such as the scanners stopping. Thus, the overall sensitivity of the system can potentially be increased. This will come, however, at the cost of increased system complexity, including the implementation of scan failure shutters, and limitations of observation times mandated by considerations of increased tissue heating from repeated light exposure.

Recent work on clinical AO-OCT imaging has outlined the importance of high resolution imaging of the retina in three dimensions^42,43^. With the instrument we introduce here, commercialization of this technology comes one step closer and possibly triggers a more widespread clinical use of such instruments. This, however, will be essential in order to fully exploit the wealth of information and details on cellular structures provided by AO-corrected imaging of the retina.

## Methods

**Figure 8:**
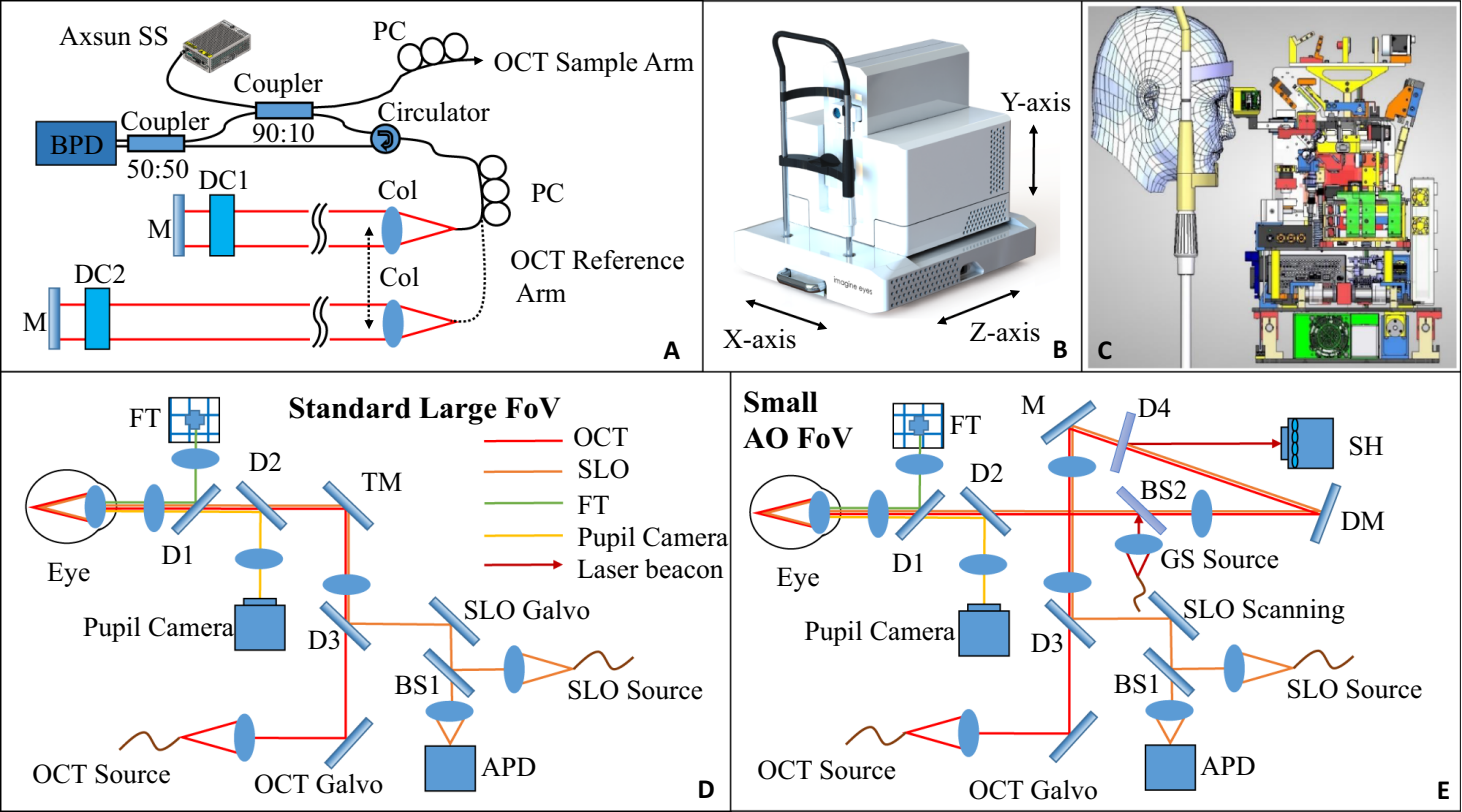
Multi-modal and multi-scale retinal imaging instrument design. (**A**) Schematic diagram of the OCT interferometer with two separate reference arms for the two imaging modes. (**B**) Three dimensional instrument design with motorized stages. (**C**) System architecture that shows the arrangement of different components inside the system. (**D**) Standard large field of view beam path for OCT, SLO, FT, pupil camera. (**E**) Beam path for small AO field of view mode with an additional laser beacon for wavefront measurement. This path requires only flipping the TM mirror. *APD* avalanche photodiode, *BPD* balanced photodetector, *BS* beam splitter, *Col* collimator, *D* dichroic mirror, *DC* dispersion compensating rods, *DM* deformable mirror, *FT* fixation target, *GS* guide star, *PC* polarization controller, *SH* Shack-Hartmann wavefront sensor.

The instrument supports two basic imaging modes: scanning laser ophthalmoscopy (SLO) and optical coherence tomography (OCT). The field of view of both imaging modes can be switched between a standard large field of view and a high-resolution, small field of view with adaptive optics (AO) correction. The schematic diagram of the instrument with SLO and OCT is shown in Fig. 8. For SLO operation, a superluminescent diode (SLD) with a central wavelength of 786 nm (bandwidth = 22 nm) is used. The image acquisition rate of 13 Hz is achieved by a resonant scanner operating at 8 kHz resulting in images containing 800 × 600 pixels. An avalanche photodiode (APD) is used to detect the back-scattered SLO light from the retina. The lateral resolution of the SLO modality is 20 µm and 3.2 µm for the standard and high resolution modes, respectively. In standard large field of view (FOV) OCT mode, pixel spacing is 10 µm while in small FOV with AO, pixel spacing is 1.5 µm and optical resolution is indeed 3.2 µm.

For OCT imaging, a swept-source (Axsun, Excelitas Technologies, USA) with a central wavelength of 1050 nm and bandwidth of 110 nm is used. The sweep rate is 200 kHz which translates to various scanning patterns. For example, a B-scan rate of 200 Hz with each B-scan containing 1000 A-scans can be achieved. The single mode fiber output of the light source with 20 mW emission power is connected to a fiber coupler with a splitting ratio of 90:10 such that 10% of the light is directed to the sample arm while 90% is directed to the reference arm of the interferometer that incorporates an optical fiber circulator (cf. Fig. 8A). In the sample arm (cf. Fig. 8D), the light is sent to the galvanometer scanners for x and y scanning of the beam. The beam then traverses a dichroic mirror that is used to combine the light beams of the SLO and the OCT modalities. Both beams then traverse a scanning lens and are reflected by a movable mirror. This mirror is intended for switching between the large field of view mode and the AO mode. In the large field of view mode, the beams traverse two dichroic mirrors that are used for the pupil camera and for displaying an internal fixation target to the subject. Finally, the beams traverse an axially displaceable final lens that collimates the beams again and that allows for adjustment of the focus within the retina.

In the AO-correction mode (cf. Fig. 8E), the movable mirror is removed from the beam path and the SLO and OCT beams traverse a dichroic mirror (used to divert the wavefront sensing light onto the Shack Hartmann wavefront sensor) and are reflected by the deformable mirror (DM, Imagine Eyes, 52 elements). The beams then traverse another dichroic mirror that is used to couple the wavefront sensing light into the system. The wavefront sensing light has the wavelength of 920 nm. The AO loop is operated at 10 Hz. Beyond that point, the beam propagation is identical to the large field of view imaging mode. The light that is backscattered from the retina propagates back the same way, and the SLO and OCT beams are separated by a dichroic mirror (D3). Both beams are de-scanned and the OCT light is coupled back into the single mode fiber while the SLO light is directed to a pinhole and detected by the APD.

The OCT light directed to the reference arm traverses a circulator, exits the fiber and is collimated by a lens. The circulator enables a double pass configuration in the bulk optics part of the reference arm and thus a shorter free space path can be realized which is required to maintain a compact instrument design. The bulk optics part (delay line) consists of a folded beam path that includes a shorter path for the large field of view mode and a longer path for the adaptive optics mode. Switching between the paths is achieved by a micro motor that vertically displaces the output collimator as shown in Fig. 8A. Each path within the delay line is terminated by mirrors that are both placed on the same translation stage for adapting the overall path length of the delay line to the variations of eye lengths between subjects. The light in the reference arm is back reflected by the mirror and is then back-coupled into the fiber of the circulator. The light is brought to interference with the light returning from the sample arm in the final 50:50 fiber beam splitter. Finally, the light is detected by two detectors that are electronically connected. This configuration allows for balanced detection of the interferometric signal and thus in a reduction of excess noise generated by the light source. The polarization controllers (PC) in the setup are used to match the polarization state of the light returning from the sample and the reference arm, respectively. For the OCT imaging modality, an axial resolution of 7 µm (in tissue) was measured. The lateral resolution is 15 µm for the large field of view mode and approximately 4.1 µm in the high-resolution small field of view AO mode.

The galvanometer scanners for the SLO and OCT imaging modalities are driven by a field programmable gate array (FPGA) that uses the trigger signal of the resonant scanner with 8 kHz frequency for SLO and the A-scan trigger of the swept source with 200 kHz frequency for OCT as respective clock signals for the corresponding voltage changes of the respective galvanometer scanners. For OCT, the fast-scanning galvanometer frequency is a function of the number of A-scans within a B-scan and is determined by the 200 kHz sweep (or A-scan) rate of the used tunable light source. For the large field of view mode we use 200 Hz B-scan rate and each B-scan consists of 1000 A-scans. It takes ~ 2 s for a volume acquisition consisting of 1000 A-scans × 400 B-scans. For AO-OCT mode (for example 300 A-scans, 300 B-scans), the recording time is reduced to ~ 0.5 s.

Retinal tracking is performed based on the SLO images. The position of the retina is determined by calculating the maximal probability of the correlation function between the acquired image and a static reference image. This process runs in parallel with the acquisition and is performed on a fast Graphical Processing Unit (GPU), allowing real time compensation of motion. The calculation is performed in the Fourier space and allows a dynamic filtering of the correlation. This results in an increased robustness of the tracking without affecting the performance of the system. At the end of the calculation, the detected position is evaluated by taking into account the acquired signal strength and the quality of the correlation image. The process provides a position value which is then sent directly to the FPGA of the OCT that adds corresponding voltage values to the driving signal of the scanners. The total measured correction latency is 10 ms (+/− 4 ms). The precision of the positioning is 2 pixels and the bandwidth is 7.4 pixels/ms.

The entire instrument is placed on a platform that can be moved by motorized stages in three dimensions (cf. Fig. 8B). For pupil tracking, standard NIR LEDs with wavelengths in the range 830–900 nm are used to illuminate the anterior segment of the eye. The reflections of the alignment LEDs on the cornea are recorded by a corresponding camera and their positions are identified. This information is used to align and track the position of the pupil during the device operation. According to the feedback, motors move the whole system to ensure that the pupil remains in the same position in respect to the instrument during the measurement.

Figure 8C shows the compactness of the instrument with all components placed inside for the optical and mechanical layout. The entire instrument has a dimension of 490 mm × 500 mm × 500 mm as shown in Fig. 8B, enclosed with the three motorized translation stages for pupil tracking of the eye which greatly simplifies subject alignment.

The overall power sent to the eye used in this study was always kept below the safety limits according to laser safety standards. For OCT imaging the light power was below 1.3 mW, for SLO imaging below 0.4 mW and the laser beacon provided 0.025 mW to the eye.

Written informed consent was obtained from all participants following an explanation of experimental procedures and risks, both verbally and in writing. All experiments were approved by the appropriate ethics review boards in France (CPP and ANSM (IDRCB number: 2019-A00942-55) and in Denmark (Videnskabsetisk Komité for Region Hovedstaden, jr. nr. H-19068888, Lægemiddelstyrelsen, jr. nr. 2020081671, EUDAMED CIV-ID nr. CIV-20-08-034465, Videnscenter for Dataanmeldelser jr. nr. P-2020–1001) and adhered to the tenets of the Declaration of Helsinki. Additional informed written consent was obtained from corresponding subjects for publishing their fundus photos in an online open access journal.

For clinical implementation of the instrument a graphical user interface (GUI) was developed. It enables entering patient information, browsing the patient list, driving the system for image acquisition and viewing acquired images. For the imaging modes, the GUI provides live visualizations for adjustments before acquisition. It enables an easy use of the multi-modal and multi-scale features as well as the AO correction for patient examination.

### Image post processing

The GPU is used for post processing of the acquired OCT data. The workstation contains the NVIDIA Quadro P2000 graphics card for fast data evaluation. Multithread GPU-based post processing of acquired data is used for visualization of the imaging results within a few seconds.

OCT angiography is achieved by acquiring four B-scans per position in both large and small fields of view. An algorithm based on amplitude decorrelation of the full spectrum is used that has been previously presented^44,45^. Each pair of successive B-scan amplitudes (A_1_ (*x,z*) and A_2_(*x,z*)) is registered to each other (using cross correlation) and the decorrelation D(*x,z*) is calculated between the two successive B-scans as follows:

1 $$D\left(x,z\right)=1-\frac{{A}_{1}(x,z){A}_{2}(x,z)}{\frac{1}{2}{\left({A}_{1}\left(x,z\right)\right)}^{2}+\frac{1}{2}{\left({A}_{2}\left(x,z\right)\right)}^{2}}$$

The total intensity I(*x,z*) at a specific location is calculated via the sum of the two B-scans:

2 $$I\left(x,z\right)={A}_{1}\left(x,z\right)+{A}_{2}\left(x,z\right)$$

In order to remove noise, pixel locations that showed values in the intensity image I(*x,z*) below a certain intensity threshold are set to zero in the decorrelation image D(*x,z*). In the final step, the intensity images I(*x,z*) resulting from the three successive pairs of B-scans are registered to each other and the same transformations are applied to the corresponding decorrelation images D(*x,z*). The resulting three decorrelation images (and three intensity images) are averaged. In the case that two B-scans that were used to calculate the decorrelation image showed poor correlation, the corresponding decorrelation image is discarded in order to enhance the OCTA data.

### Image registration

Intensity-based pairwise registration based on the Elastix toolbox^46^ is applied for motion correction in all the imaging modalities (SLO/OCT/OCTA) and both fields of view (large and AO). Pairs of 2D images are aligned iteratively in a multi-resolution scheme, that is, down-sampling and smoothing each image several times, so that the initial alignment is guided by the roughest details and the final refinement is computed in the original data. The transformation parameters for each pair are computed by minimizing a normalized correlation metric, using a stochastic gradient descent optimizer.

For each OCT 3D acquisition, two volumes are obtained. If any of the volumes is free of blinks, that volume is used for further processing. Otherwise, the B-scans from the second volume are used to fill in the missing information at the blinks, obtaining a complete 3D dataset. Each B-scan of this resulting volume is spatially aligned to the previous one, allowing only translations (in both the axial and lateral directions). To avoid the over-correction effect of the registration, the computed translations are smoothed, so that only the sudden spikes caused by the motion are corrected. For the OCTA data, the translations are computed following this same approach in the paired OCT data, and then applied to the OCTA.

A similar registration pipeline is used to obtain the high quality OCT images resulting from averaging of several B-scans acquired at the same position. The individual B-scans are first sorted, so that the ones that are the least similar (e.g. due to blinks or strong artifacts) to the mean intensity of the set are discarded. The remaining B-scans are registered iteratively, allowing rotations and translations in both directions (axial and lateral). After averaging the co-localized B-scans, the resulting image is displayed in log scale and after intensity normalization.

In order to generate high quality averaged SLO data, the initial set of images is also sorted, ensuring that images showing strong artifacts are not included. The individual SLO images may have larger deformations than the B-scans and, hence, more freedom is allowed in the transformation model. The large field of view data uses affine transformations, while the AO data uses local deformations following a B-spline grid.

Finally, software-based co-localization is applied to ensure that the AO image is displayed at the real position in the large field of view scan. To that end, the AO data of the different modalities is matched to the large field of view OCT volume using the structural similarity index metric^47^:

3 $$SSIM(a,b) = \frac{(2{\mu }_{a}{\mu }_{b} +{c}_{1})(2{\sigma }_{ab} +{c}_{2})}{({{\mu }_{a}}^{2}{ + {\mu }_{b}}^{2} +{c}_{1})({{\sigma }_{a}}^{2}{ + {\sigma }_{b}}^{2} +{c}_{2})}$$

where $a$ and $b$ correspond to the AO image and OCT region of interest, respectively, $\mu$ is the average;$\sigma$, the variance; ${\sigma }_{ab}$, the covariance of $a$ and $b$; and ${c}_{1}$ and ${c}_{2}$ are two variables to stabilize the division in case of near-zero denominators.

## Conclusion

The multiscale architecture of the new device for imaging of the fundus of the eye enables large-field SLO, OCT and OCTA examinations for rapid identification of regions of interest, combined with a high-magnification mode that provides the same three imaging modalities under AO correction for a selected subfield. The capacity to visualize individual cells in three dimensions, as well as the ability to image both the structure and the perfusion of retinal blood vessels down to the capillary level will enable a wide array of new applications in a compact instrument that suits existing outpatient clinical environments. With the proposed device, healthy and pathological characteristics of the retina, known only from rare histopathological tissue examination, can potentially be examined non-invasively in vivo. Potential applications include refined diagnostics, guidance and monitoring of treatment and the development of new treatment modalities.

## Data Availability

The datasets generated during and/or analysed during the current study are available from the corresponding author on reasonable request.

## References

[1] Webb RH, Hughes GW, Delori FC (1987). Confocal scanning laser ophthalmoscope. Appl. Opt..

[2] Huang D (1991). Optical coherence tomography. Science.

[3] Makita S, Hong Y, Yamanari M, Yatagai T, Yasuno Y (2006). Optical coherence angiography. Opt. Express.

[4] de Carlo TE, Romano A, Waheed NK, Duker JS (2015). A review of optical coherence tomography angiography (OCTA). Int. J. Retina Vitreous.

[5] Gao SS (2016). Optical coherence tomography angiography. Investig. Ophthalmol. Vis. Sci..

[6] Ferrara D, Waheed NK, Duker JS (2016). Investigating the choriocapillaris and choroidal vasculature with new optical coherence tomography technologies. Prog. Retin. Eye Res..

[7] Jonnal RS (2016). A review of adaptive optics optical coherence tomography: Technical advances, scientific applications, and the future. Invest. Ophthalmol. Vis. Sci..

[8] Merino D, Loza-Alvarez P (2016). Adaptive optics scanning laser ophthalmoscope imaging: Technology update. Clin. Ophthalmol..

[9] Pircher M, Zawadzki RJ (2017). Review of adaptive optics OCT (AO-OCT): Principles and applications for retinal imaging [Invited]. Biomed. Opt. Express.

[10] Bakker E (2021). Adaptive optics ophthalmoscopy: A systematic review of vascular biomarkers. Surv. Ophthalmol..

[11] Roorda A (2002). Adaptive optics scanning laser ophthalmoscopy. Opt. Express.

[12] Zhang Y (2006). High-speed volumetric imaging of cone photoreceptors with adaptive optics spectral-domain optical coherence tomography. Opt. Express.

[13] Burns SA, Tumbar R, Elsner AE, Ferguson D, Hammer DX (2007). Large-field-of-view, modular, stabilized, adaptive-optics-based scanning laser ophthalmoscope. J. Opt. Soc. Am. A.

[14] Kocaoglu OP (2011). Imaging cone photoreceptors in three dimensions and in time using ultrahigh resolution optical coherence tomography with adaptive optics. Biomed. Opt. Express.

[15] Dubra A (2011). Noninvasive imaging of the human rod photoreceptor mosaic using a confocal adaptive optics scanning ophthalmoscope. Biomed. Opt. Express.

[16] Laslandes M, Salas M, Hitzenberger CK, Pircher M (2017). Increasing the field of view of adaptive optics scanning laser ophthalmoscopy. Biomed. Opt. Express.

[17] Shirazi MF (2020). Visualizing human photoreceptor and retinal pigment epithelium cell mosaics in a single volume scan over an extended field of view with adaptive optics optical coherence tomography. Biomed. Opt. Express.

[18] Torti C (2009). Adaptive optics optical coherence tomography at 120,000 depth scans/s for non-invasive cellular phenotyping of the living human retina. Opt. Express.

[19] Felberer F (2014). Adaptive optics SLO/OCT for 3D imaging of human photoreceptors in vivo. Biomed. Opt. Express.

[20] Liu Z, Kocaoglu OP, Miller DT (2016). 3D imaging of retinal pigment epithelial cells in the living human retina. Investig. Ophthalmol. Vis. Sci..

[21] Liu Z, Kurokawa K, Zhang F, Lee JJ, Miller DT (2017). Imaging and quantifying ganglion cells and other transparent neurons in the living human retina. Proc. Natl. Acad. Sci..

[22] McGregor JE (2020). Optogenetic restoration of retinal ganglion cell activity in the living primate. Nat. Commun..

[23] Bigelow CE (2007). Compact multimodal adaptive-optics spectral-domain optical coherence tomography instrument for retinal imaging. J. Opt. Soc. Am. A.

[24] Mujat M (2010). High resolution multimodal clinical ophthalmic imaging system. Opt. Express.

[25] Hammer DX (2012). Multimodal adaptive optics retinal imager: Design and performance. J. Opt. Soc. Am. A.

[26] Jian Y (2016). Lens-based wavefront sensorless adaptive optics swept source OCT. Sci. Rep..

[27] Salas M (2016). Multi-modal adaptive optics system including fundus photography and optical coherence tomography for the clinical setting. Biomed. Opt. Express.

[28] Bower AJ (2021). Integrating adaptive optics-SLO and OCT for multimodal visualization of the human retinal pigment epithelial mosaic. Biomed. Opt. Express.

[29] Salas M (2018). Compact akinetic swept source optical coherence tomography angiography at 1060 nm supporting a wide field of view and adaptive optics imaging modes of the posterior eye. Biomed. Opt. Express.

[30] Hammer DX (2006). Adaptive optics scanning laser ophthalmoscope for stabilized retinal imaging. Opt. Express.

[31] Vienola KV (2012). Real-time eye motion compensation for OCT imaging with tracking SLO. Biomed. Opt. Express.

[32] Kocaoglu OP (2014). Adaptive optics optical coherence tomography with dynamic retinal tracking. Biomed. Opt. Express.

[33] Sahin B, Lamory B, Levecq X, Harms F, Dainty C (2012). Adaptive optics with pupil tracking for high resolution retinal imaging. Biomed. Opt. Express.

[34] de Castro A, Sawides L, Qi X, Burns SA (2017). Adaptive optics retinal imaging with automatic detection of the pupil and its boundary in real time using Shack–Hartmann images. Appl. Opt..

[35] Lujan BJ, Roorda A, Knighton RW, Carroll J (2011). Revealing Henle's fiber layer using spectral domain optical coherence tomography. Invest. Ophthalmol. Vis. Sci..

[36] Jonnal RS (2014). The cellular origins of the outer retinal bands in optical coherence tomography images. Invest. Ophthalmol. Vis. Sci..

[37] Cuenca N, Ortuño-Lizarán I, Pinilla I (2018). Cellular characterization of OCT and outer retinal bands using specific immunohistochemistry markers and clinical implications. Ophthalmology.

[38] Srinivasan VJ (2008). Characterization of outer retinal morphology with high-speed, ultrahigh-resolution optical coherence tomography. Invest. Ophthalmol. Vis. Sci..

[39] Willerslev A, Li XQ, Cordtz P, Munch IC, Larsen M (2014). Retinal and choroidal intravascular spectral-domain optical coherence tomography. Acta Ophthalmol..

[40] Munk MR (2017). OCT-angiography: A qualitative and quantitative comparison of 4 OCT-A devices. PLoS ONE.

[41] Salas M (2017). Visualization of micro-capillaries using optical coherence tomography angiography with and without adaptive optics. Biomed. Opt. Express.

[42] Reumueller A (2019). Three-dimensional adaptive optics-assisted visualization of photoreceptors in healthy and pathologically aged eyes. Invest. Ophthalmol. Vis. Sci..

[43] Reumueller A (2020). Three-dimensional assessment of para- and perifoveal photoreceptor densities and the impact of meridians and age in healthy eyes with adaptive-optics optical coherence tomography (AO-OCT). Opt. Express.

[44] Jia YL (2012). Split-spectrum amplitude-decorrelation angiography with optical coherence tomography. Opt. Express.

[45] Spaide RF, Fujimoto JG, Waheed NK, Sadda SR, Staurenghi G (2018). Optical coherence tomography angiography. Prog. Retin. Eye Res..

[46] Klein S, Staring M, Murphy K, Viergever MA, Pluim JP (2010). elastix: A toolbox for intensity-based medical image registration. IEEE Trans Med Imaging.

[47] Zhou W, Bovik AC, Sheikh HR, Simoncelli EP (2004). Image quality assessment: From error visibility to structural similarity. IEEE Trans. Image Process..

